# Supplementary material for: Microwave Irradiation vs. Structural, Physicochemical, and Biological Features of Porous Environmentally Active Silver–Silica Nanocomposites
Source: Int J Mol Sci. 2023 Apr 1;24(7):6632. doi: 10.3390/ijms24076632 (PMC10095382; doi:10.3390/ijms24076632)
Supplement: Supplementary file 1 [file ijms-24-06632-s001.zip › ijms-2164624-supplementary.pdf]

Supplementary data

Microwave irradiation vs structural, physicochemical and biological features of porous environmentally active silver-silica nanocomposites

Aleksandra Strach [1], Mateusz Dulski [2,*], Daniel Wasilkowski [3], Oliwia Metryka [1], Anna Nowak [3], Krzysztof Matus [4], Karolina Dudek [5], Patrycja Rawicka [6], Jerzy Kubacki [6], Natalia Waloszczyk [7], Agnieszka Mrozik [3] and Sylwia Golba [2]

[1] Doctoral School, University of Silesia, Bankowa 14, 40-032 Katowice, Poland; aleksandra.strach@us.edu.pl (A.S.); oliwia.metryka@us.edu.pl (O.M.)
[2] Institute of Materials Engineering, and Silesian Center for Education and Interdisciplinary Research, 75 Pulku Piechoty 1A, 41-500 Chorzow, Poland; sylwia.golba@us.edu.pl
[3] Institute of Biology, Biotechnology, and Environmental Protection, Faculty of Natural Sciences, University of Silesia, Jagiellońska 28, 40-032 Katowice, Poland; daniel.wasilkowski@us.edu.pl (D.W.); anna.m.nowak@us.edu.pl (A.N.); agnieszka.mrozik@us.edu.pl (A.M.)
[4] Materials Research Laboratory, Silesian University of Technology, Konarskiego 18A, 44-100 Gliwice, Poland; krzysztof.matus@polsl.pl
[5] Łukasiewicz Research Network, Institute of Ceramics and Building Materials, Cementowa 8, 31-938 Cracow, Poland; karolina.dudek@icimb.lukasiewicz.gov.pl
[6] A. Chełkowski Institute of Physics, University of Silesia, 75 Pulku Piechoty 1, 41-500 Chorzow, Poland; patrycja.rawicka@us.edu.pl (P.R.); jerzy.kubacki@us.edu.pl (J.K.)
[7] Faculty of Chemistry, Silesian University of Technology, 44-100 Gliwice, Poland; natalia.szulc@polsl.pl
* Correspondence: mateusz.dulski@us.edu.pl

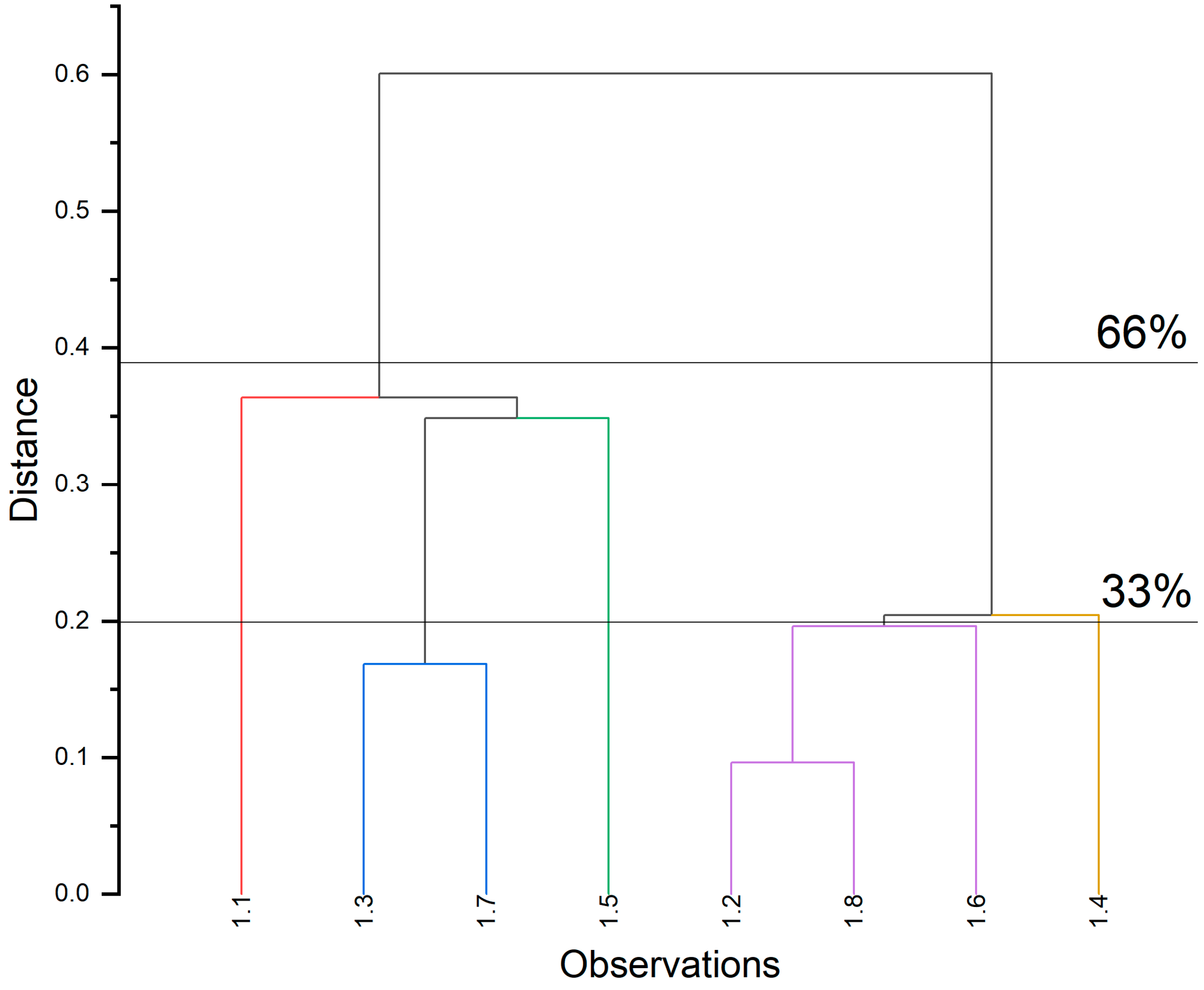

Figure S1. Hierarchical cluster analysis performed between SSA_{BET} and D_{pore} with Sneath criteria grouped samples Ag-S1 and Ag-S2 into individual clusters.

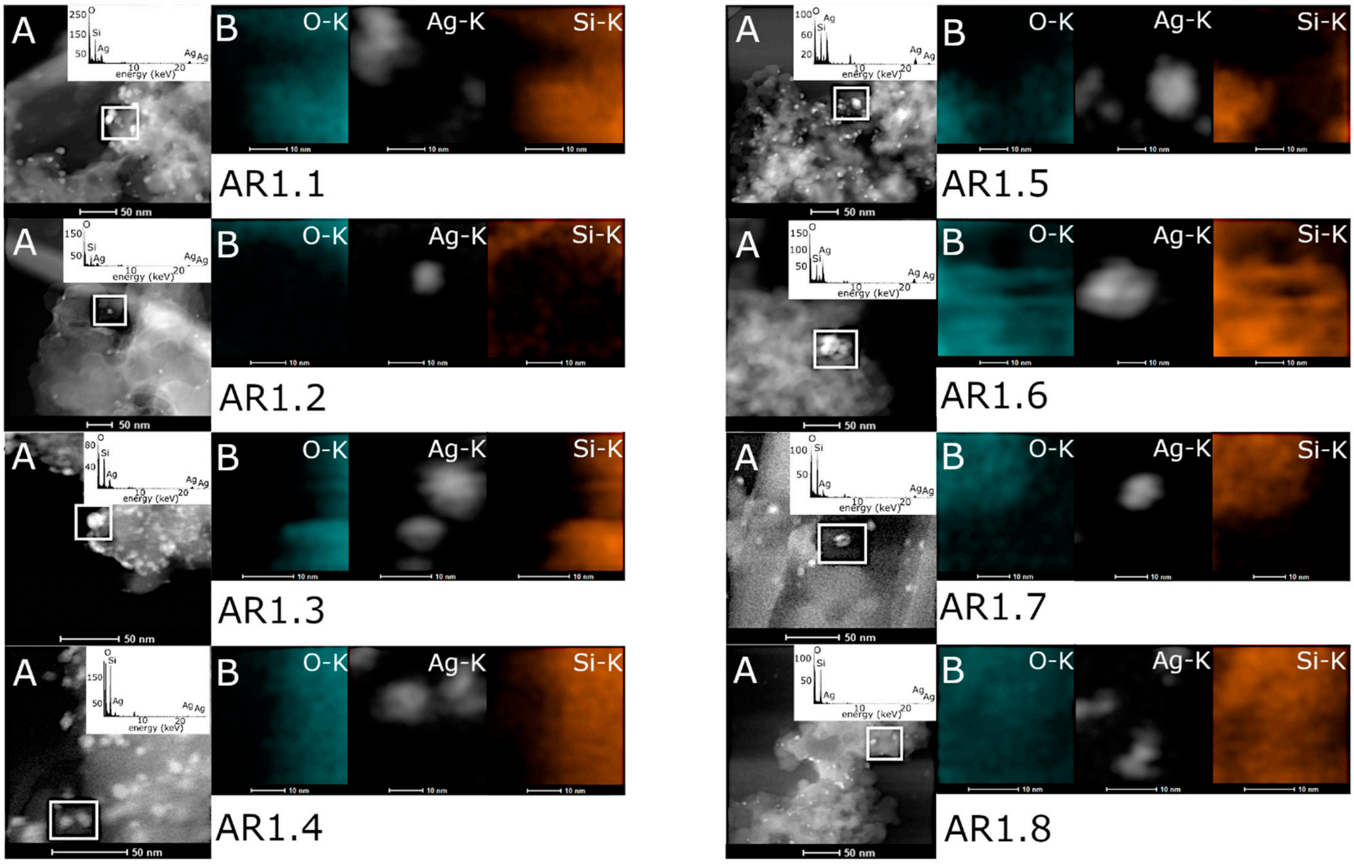

Figure S2. (A) HRTEM micro images with TEM-EDS spectrum in the inset. (B) HRTEM chemical mapping around the white-rectangular highlighted place performed for Ag-SiO_2 nanocomposites (left panels refer to S1 Silica, right panel to S2 Silica). .
